# Membrane heat shock protein 70: a theranostic target for cancer therapy

**DOI:** 10.1098/rstb.2016.0526

**Published:** 2017-12-04

**Authors:** Maxim Shevtsov, Gao Huile, Gabriele Multhoff

**Affiliations:** 1 Klinikum rechts der Isar, Department of Radiation Oncology, Technische Universität München, Ismaninger Strasse 22, Munich 81675, Germany; 2 Institute of Cytology of the Russian Academy of Sciences, Tikhoretsky Avenue, 4, St Petersburg 194064, Russia; 3 West China School of Pharmacy, Sichuan University, Chengdu 610041, People's Republic of China

**Keywords:** cancer, theranostic, nanoparticle therapy, HSP70 targeting

## Abstract

Members of the 70 kDa stress protein family are found in nearly all subcellular compartments of nucleated cells where they fulfil a number of chaperoning functions. Heat shock protein 70 (HSP70), also termed HSPA1A, the major stress-inducible member of this family is overexpressed in a large variety of different tumour types. Apart from its intracellular localization, a tumour-selective HSP70 membrane expression has been determined. A membrane HSP70–positive tumour phenotype is associated with aggressiveness and therapy resistance, but also serves as a recognition structure for targeted therapies. Furthermore, membrane-bound and extracellularly residing HSP70 derived from tumour cells play pivotal roles in eliciting anti-tumour immune responses. Herein, we want to shed light on the multiplicity of different activities of HSP70, depending on its intracellular, membrane and extracellular localization with the goal to use membrane HSP70 as a target for novel therapies including nanoparticle-based approaches for the treatment of cancer.

This article is part of the theme issue ‘Heat shock proteins as modulators and therapeutic targets of chronic disease: an integrated perspective’.

## 1. Role of intracellular, membrane and extracellular heat shock protein 70 in tumour cells

Members of the 70 kDa chaperone family are found in nearly all subcellular compartments of nucleated cells [1], where they support folding of nascent polypeptides, prevent protein aggregation and assist transport of proteins across membranes [2,3]. The major stress-inducible HSP70, also termed HSPA1A, is frequently overexpressed in a large variety of different tumour types [4]. In comparison to other stress proteins of the HSP70 group, the synthesis of HSP70 is more rapid, and HSP70 accumulates at higher levels in tumour cells after exposure to environmental stress. High cytosolic HSP70 levels are known to protect cancer cells from apoptotic cell death, promote tumour cell proliferation and migration, mediate therapy resistance and thus contribute to an aggressive tumour phenotype. Therefore, the reduction or inhibition of intracellular HSP70 levels by different methods such as shRNA, CRISPR/Cas9 knock-out technology, aptamers or HSP70 inhibitors [5] provides a promising strategy to sensitize tumour cells towards therapy by antagonizing apoptosis and inducing Bcl-2/caspase-independent cell death [6–8]. However, due to redundancy of the chaperone system in eukaryotic cells, other stress proteins can take over tasks of HSP70 and thereby limit efficacy of HSP70-depleting approaches. Apart from the cytosol, HSP70 also resides within lysosomal membranes of tumour cells which in turn mediate resistance to cell death by membrane stabilization [9]. Interference with the sphingolipid metabolism of lysosomal membranes has shown promising results in breaking therapy resistance in tumour mouse models [10]. As to whether this strategy might be transferable into the human system remains to be determined.

Global proteome profiling of plasma membrane–bound proteins reveals an abundance of cytosolic chaperones, including HSP70, on the surface of tumour cells [11]. Although most cytosolic stress proteins typically lack a classical consensual transmembrane sequence, a tumour-specific cell surface localization of HSP70 could be determined by different methods, including multi-parameter flow cytometry with an antibody (cmHsp70.1) that specifically recognizes the membrane conformation of HSP70 [12], and selective cell surface iodination of membrane proteins [13]. Despite a membrane HSP70 positivity in many different tumour types, several questions remain to be answered: how are cytosolic HSPs transported from the cytosol to the plasma membrane, how are they anchored in the plasma membrane, and why is this phenomenon restricted to malignantly transformed cells. As inhibition of the post-Golgi membrane traffic by Brefeldin A (BFA), a lacton antibiotic and monensin are unable to block the plasma membrane transport of HSP70, a classical ER-Golgi transport mechanism appears to be unlikely [14]. Currently, it is assumed that HSP70 is transported to the plasma membrane via non-classical mechanisms mediated by intracellular lipid vesicles. As modification of extracellular salt concentration and pH does not alter the membrane density of HSP70, it is not very likely that membrane HSP70 interacts with proteineous surface receptors [15]. Already in 1989, Hightower & Guidon [14] suggested a direct association of HSP70 with lipid components. This hypothesis is reasonable because HSP70 shows a high affinity for hydrophobic/lipophilic regions of unfolded proteins.

At present, different mechanisms are discussed as to how HSP70 might interact with lipid components of the plasma membrane of tumour cells. As demonstrated by interaction studies with artificial lipid nanovesicles, HSP70 was shown to directly interact with tumour-specific lipids such as globoyltriaosylceramide (Gb3) [15]. As Gb3 is a typical component of cholesterol-rich microdomains, also termed lipid rafts [16], it is assumed that HSP70 might reside in lipid rafts of the plasma membrane of tumour cells. Lipid rafts serve as assembly and sorting platforms for signal transduction and, therefore, the molecular chaperone HSP70 might be required for supporting signalling and cross-talk between different tumour cells [17]. This assumption has been supported by the finding that the destruction of lipid rafts by cholesterol depletion using methyl-β-cyclodextrin results in a loss of membrane HSP70 [15].

Although it is still a matter of debate whether membrane-bound HSP70 exerts chaperoning activity for adjacent proteins, such as receptors or signalling molecules, a membrane localization of HSP70 has been associated with different diseases, including neurotoxic prion disease [18], encephalitis viral diseases [19], malaria-infected erythrocytes [20,21] and glioblastomas [22].

Apart from Gb3, as one example for a potential interaction partner for HSP70 in the plasma membrane, a physical interaction of HSP70 also has been determined for phosphatidylserine (PS) a lipid that is not found in lipid rafts. Under non-stressed conditions, PS is exclusively located in the inner leaflet of the plasma membrane. The asymmetry of PS is maintained by the ATP-dependent aminophospholipid translocase [23]. An activation of the Ca^2+^-dependent phospholipid scramblase and by ATP depletion results in a loss of the PS lipid asymmetry [24]. The appearance of PS in the outer membrane leaflet is considered as an early marker of apoptosis [25] that can trigger recognition of dying cells by macrophages [26]. However, CD8^+^ T cells undergoing antigen recognition have been shown to present PS on their outer membrane leaflet without undergoing apoptotic cell death [27]. In viable tumour cells, PS also might be considered as an interacting partner for HSP70 in the plasma membrane. In line with this hypothesis, we and others have shown that HSP70 preferentially inserts into artificial unilamellar lipid vesicles containing high levels of PS [28,29]. This affinity of HSP70 to PS suggests that HSP70 might travel from the inner to the outer membrane leaflet through a translocation of PS. De Maio and co-workers [30] suggested that several HSP70 proteins can form ion conductance channels within artificial lipid bilayers containing PS. This group also showed that HSP70 preferentially interacts with negatively charged phospholipids within liposomes, which enable not only the insertion of high molecular–weight HSP70 complexes in lipid bilayer membranes, but also enable an ER-Golgi–independent export of HSP70. Along this line, our group demonstrated that exogenously applied HSP70 protein preferentially interacts with PS on the outer membrane leaflet. This interaction of HSP70 form outside can promote tumour cell killing, especially under stress [29]. In a pilot study with glioblastoma patients, an intra-tumoural injection of HSP70 protein was found to trigger tumour cell death [22]. Although the mode of action of this therapeutic approach has not yet been elucidated, the interaction of external HSP70 with the plasma membrane of tumour cells appears to initiate this process. The authors of this pilot study speculate about an involvement of immune effector cells that might be able to recognize membrane HSP70.

Following therapeutic interventions such as radiochemotherapy, the cell surface density of HSP70 is further enhanced selectively on tumour cells [31,32]. Regarding this finding, membrane HSP70 also might serve as a biomarker for monitoring outcome and as a tumour-specific target for the cytolytic attack of immune cells and tumour-specific targeting molecules. Membrane HSP70 on highly aggressive tumour cells has been found to act as a trigger factor for CD56^bright^/CD94^+^ natural killer (NK) cells in the presence of pro-inflammatory cytokines [33–35]. Incubation of human NK cells with HSP70 protein or a peptide derived from the C-terminal domain of HSP70 [36,37] in combination with pro-inflammatory cytokines such as interleukin-2 (IL-2) or IL-15 has been found to activate CD56^bright^/CD94^+^ NK cells that are able to recognize and kill membrane HSP70–positive tumour cells *in vitro* and in tumour mouse models [36,37]. Based on these findings, a phase II clinical trial was initiated with the goal to determine the efficacy of *ex vivo* HSP70 peptide plus IL-2 stimulated autologous NK cells in patients with inoperable non-small cell lung cancer (NSCLC) in stage IIIA/B after radiochemotherapy [38,39]. It has been shown that HSP70-reactive NK cells can be generated reproducible from leukapheresis product of NSCLC patients and that the adoptive transfer of these activated cells is well tolerated. Furthermore, NK cell activity against membrane HSP70–positive tumour cells which initially was found to be very low in all tumour patients could be re-stimulated by an *ex vivo* stimulation with HSP70 peptide plus IL-2 as a growth factor. It is assumed that the NSCLC tumours and the tumour microenvironment induces an immunosuppressive milieu for immunocompetent effector cells. Therefore, an *ex vivo* stimulation of NK cells might be superior to a direct vaccination of patients with HSP70 peptide plus IL-2 to overcome the immunosuppressive milieu. It is expected that reactivation of the cytolytic activity of NK cells as the first line of defence is able to stimulate protective anti-tumour immunity by T cells in a second step.

Although membrane-bound HSP70 can be considered as a recognition structure for the innate immune system, tumour cells presenting HSP70 on their cell surface show a higher resistance to radiochemotherapy compared with their membrane HSP70-negative counterparts [40]. Therefore, membrane HSP70 as a tumour-specific target for immune cells has to be considered with care. An upregulated membrane HSP70 expression on tumour cells might help NK cells to recognize their target cells, however, also mediates resistance of tumour cells towards standard therapies.

Lastly, an extracellular localization of free or lipid-bound HSP70 has to be considered with respect to its functionality. In general, serum HSP70 levels in patients with different malignancies including tumours and infectious diseases are higher than those of healthy individuals. It has been shown that tumour patients exhibit significantly higher levels of serum HSP70 than patients with infectious disease [41].

Free HSP70 is generally assumed to originate from dying cells, whereas lipid-bound, vesicular HSP70 appears to be actively released from viable tumour cells [28,42,43]. Physico-chemical analysis of these extracellular HSP70-bearing lipid vesicles characterized them as exosomes. The exosomal release is explained by an alternative lysosomal/endosomal pathway which does not involve the ER-Golgi compartment. Depending on the microRNA and protein composition which is present in the lumen and on the surface, actively released tumour exosomes either mediate stimulatory or inhibitory anti-tumour immune responses. Exosomes with a diameter of 40–100 nm and defined floating characteristics (1.17–1.19 g ml^−1^) are considered as soluble signalling platforms that can promote tumour growth, migration and invasion [43]. Apart from tumour cells [42,44], exosomes are also released by a large variety of other cell types including antigen-presenting cells [45], T cells, reticulocytes [46,47], B cells [48,49], platelets [50] and glia cells [51]. HSP70 released by normal human monocytes upon stimulation can prevent the formation of gap-junctions and thus can hinder intercellular communications. In turn, these complex interactions of exosomes with cells (tumour and immune cells) can also affect inflammation and tumour growth [52].

Free HSP70 that predominantly originates from dying cells also has been found to exert immunomodulatory activities, especially if immunogenic peptides are bound to them. A number of C-type lectin receptors such as LOX-1, SR-A SREC have been proposed to be involved in the uptake of chaperone–peptide complexes [53–57]. Following cross-presentation of HSP70-chaperoned peptides on MHC class I molecules, a CD8^+^ antigen-specific cytotoxic T cell response can be initiated [58–60].

The interaction of peptide-free HSP70 with CD14, a glycophosphatidylinositol GPI-anchored receptor and or TLR2/4 on antigen-presenting cells has been found to initiate the release of pro-inflammatory cytokines via NF-κB signalling [52,61]. This process has been described as the ‘chaperokine’ effect. However, at present, this concept has not yet been tested in preclinical and clinical trials.

Another mechanism, how extracellular HSP70 might affect tumour cells is the complex formation of the innate immunity protein Tag7 with HSP70 [62,63]. It has been shown that interaction of the Tag7–HSP70 complex with TNFR1 triggers the activation of RIP1-kinase, an increase in intracellular concentration of Ca^2+^ ions and an activation of calpains which result in the permeabilization of lysosomal membranes [63–66]. The lysosome-induced release of cathepsines B and D can in turn depolarize mitochondrial membranes and induce ROS production which might initiate tumour cell necroptosis.

**Figure 1.**
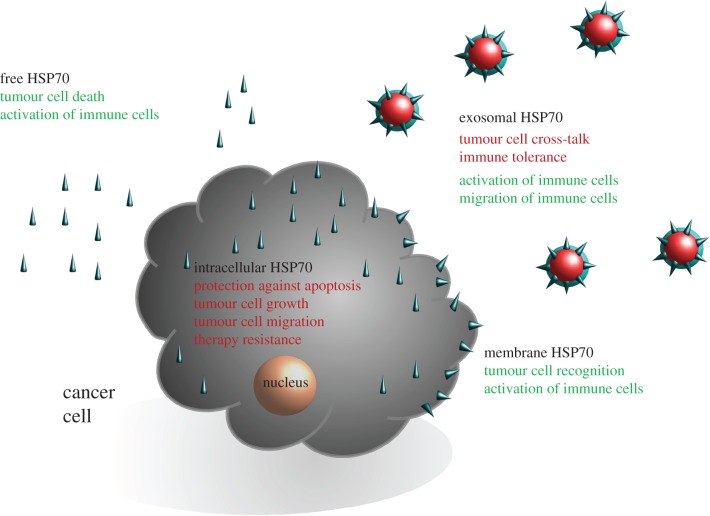
Schematic representation of the different functions of HSP70 in the cytosol, on the plasma membrane and in the extracellular space as free and exosomal HSP70. Green indicates anti-tumoural activities; red indicates pro-tumoural activities.

As summarized in figure 1, depending on its localization in the cytosol, on the membrane and outside of tumour cells, HSP70 fulfils different functions that can impact on tumour cell resistance, as well as on the stimulation of immune responses against cancer. For future clinical applications, it might be important not only to determine the total amount, but also the subcellular or extracellular localization of HSP70.

## 2. Ferromagnetic and gold nanoparticle-based therapies targeting membrane heat shock protein 70

**Figure 2.**
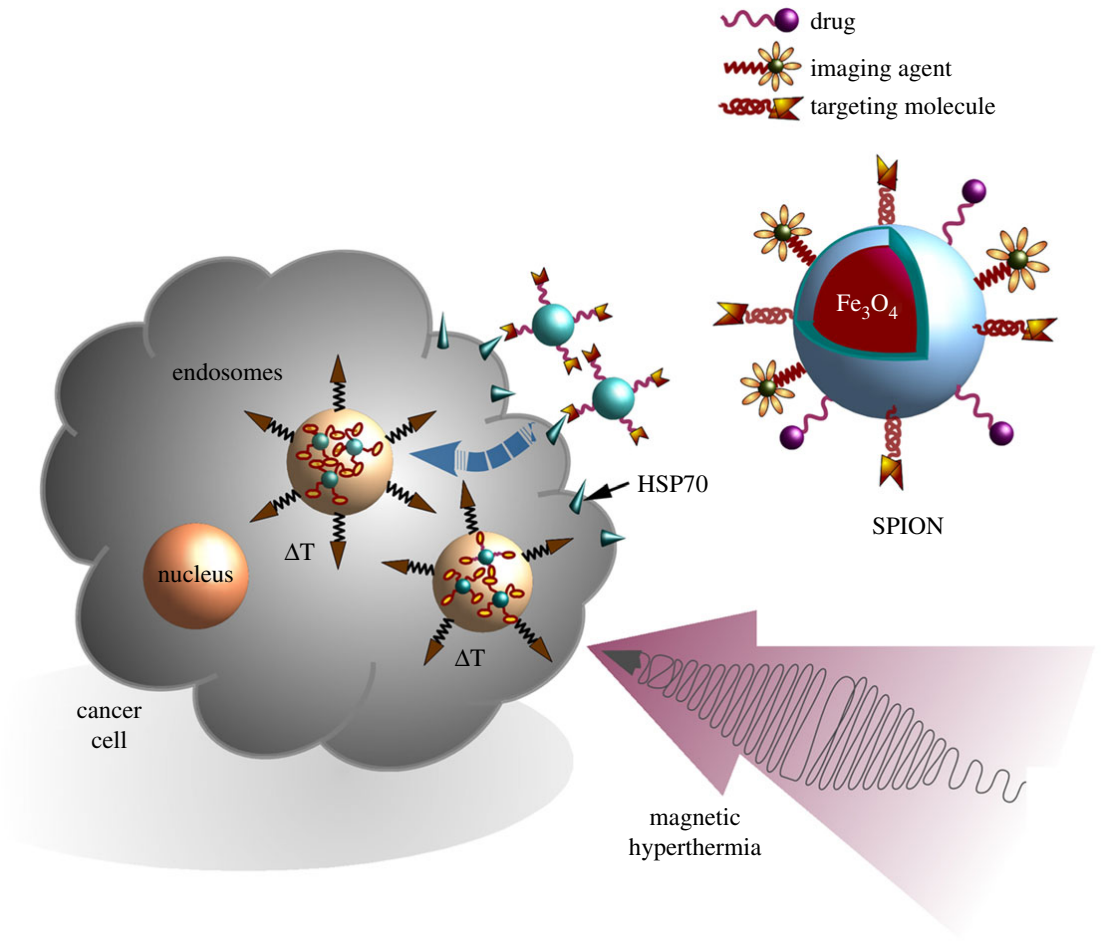
Membrane HSP70–targeting SPIONs in combination with hyperthermia for theranostics. MNPs decorated with diagnostic and/or therapeutic agents can be administered intravenously or injected directly into the tumour. Subsequent application of the applied AMF increases intra-tumoural temperature that could result in the heat-induced damage of cancer cells.

Nanoparticles provide unique multi-functional vehicles for theranostic purposes in oncology because they can remotely and non-invasively be used as imaging agents as well as carriers for a targeted drug delivery (figure 2). Different formulations, qualities and compositions of nanoparticles define their physico-chemical properties as well as their potential application in anti-cancer therapy. Magnetic nanoparticles (MNPs) based on iron oxide are one of the most widespread formulations [67]. For MNPs, several types of iron oxides, including magnetite (Fe_3_O_4_), haematite (α-Fe_2_O_3_) and maghemite (γ-Fe_2_O_3_ and β-Fe_2_O_3_) are currently used [68]. Owing to their unique magnetic and optical characteristics, such as high paramagnetism, magnetic coercivity, magnetic susceptibility and low Curie temperature, superparamagnetic iron oxide nanoparticles (SPIONs) show superior characteristics compared with other types of MNPs [69]. Previously, it was demonstrated that SPIONs with a biocompatible surface coverage are well tolerated and thus can be applied for tumour targeting *in vivo* (figure 2). The modulation of the surface of SPIONs is key for a tumour-specific targeting and drug delivery, as well as for biocompatibility and sustainability of MNPs *in vivo*. Recent studies demonstrated that surface functionalization of nanoparticles with proteins (i.e. IL-1Ra, EGF etc.) that are able to target ligands which are frequently overexpressed on tumour cells significantly increases their retention inside the tumour and improves magnetic resonance imaging, especially in the case of brain tumours [70–73]. HSP70, which is known to be expressed selectively on the surface of tumour, but not on corresponding normal cells [13,15], provides a promising targeting structure for nanoparticle-based therapies. Thus, decoration of MNPs with HSP70-targeting molecules (e.g. antibodies, peptides, Fab fragments, anticalines, etc.) could potentiate the tumour-targeting properties of these nanocarriers. Recently, it was demonstrated that the application of cmHsp70.1 antibody–functionalized SPIONs significantly increases the retention of nanoparticles in the tumour in a model of intracranial C6 glioma in rodents [71]. A subsequent ionizing irradiation that enhances the expression density of HSP70 on the plasma membrane of glioma cells resulted in an increased enrichment of HSP70-targeting particles inside the tumour [74]. Owing to their smaller size and improved biocompatibility, the application of smaller molecules such as Hsp70-reactive peptides, Fab fragments or anticalines might be able to further improve the targeting abilities of MNPs. Furthermore, application of alternating magnetic field (AMF) could convert the diagnostic potential of nanoparticles into theranostics. MNP-mediated hyperthermia (figure 2), which is based on three independent mechanisms such as hysteresis loss, Brownian and Néel relaxation, can result in thermal energy upon a magnetic and heat stimulation [75].

**Figure 3.**
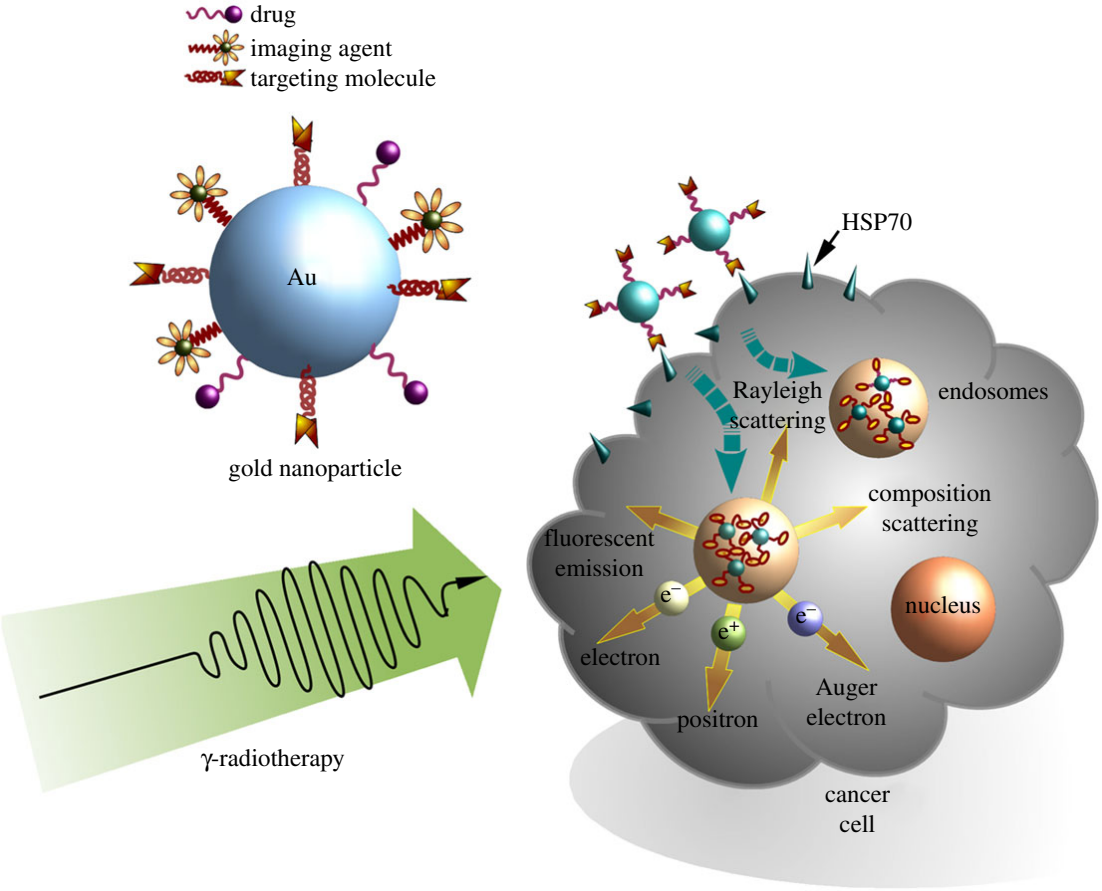
Membrane HSP70–targeting gold nanoparticles (AuNPs) in combination with ionizing irradiation for tumour therapy. Functionalization of AuNPs with tumour-targeting ligands increases the tumour-targeting potential of these nanoparticles. Sensitization of tumour cells against ionizing irradiation is attributed to the production of low energy (Auger and Coster–Kronig) electrons, secondary electrons, MV photons through Compton scattering and Rayleigh scattering.

Alternative to HSP70-targeting magnetite-based nanoparticles, a multi-modal therapy concept could also be based on gold nanomaterials, which are also widely used in cancer therapy. Gold nanoparticles (AuNPs) show an excellent biocompatibility, can be easily synthesized in a wide range of different sizes and shapes, and their surfaces can be coated with different ligands (figure 3). Therefore, AuNPs are available in many different formulations, including nanoparticles, nanorods, nanoclusters, nanoshells, nanocages, etc. [76]. Owing to the relatively high costs of gold nanomaterials, these nanoparticles are also produced as hybrids together with cheaper materials. As an example Au–gelatin–NP hybrids can be easily changed in size and thus show an improved tumour distribution and penetration [77,78]. Another study tested branched palladium nanostructures that were covered with gold, which in turn displayed a very good photothermal property in cancer therapy [79]. Currently, gold nanomaterials are used for the delivery of chemotherapeutics, proteins, genes [80–82], for photothermal [83,84] and photodynamic therapy [85], photoacoustic imaging [86], computed tomography [87] and theranostic purposes [77,78,81,86]. All these applications can be further improved by coating nanoparticles with targeting reagents such as HSP70 (figure 3). In our group, we could demonstrate that coupling of the membrane HSP70–specific antibody (cmHsp70.1) on AuNPs (AuNPs-HSP70) could significantly enhance targeting and specific uptake of nanoparticles into membrane HSP70–positive mouse colon carcinoma (CT26) cells, *in vitro* [88]. Interestingly, HSP70-coated AuNPs are found to be enriched in close proximity to the nucleus after 24 h, whereas AuNPs coated with an isoptype-matched control antibody did enter tumour cells at lower numbers and showed a more disperse intracellular distribution. This finding might be of importance with respect to future combined therapeutic approaches consisting of AuNPs and radiotherapy in the context of radioenhancing effects induced by Auger electrons. In an HSP70 knock-out mouse mammary carcinoma model, the HSP70 specificity of the uptake of HSP70-targeting AuNPs could be confirmed [88]. In summary, these data provide a first hint that the enhanced cellular uptake of HSP70-targeting AuNPs is attributed to membrane HSP70 on tumour cells.

It is well accepted that HSPs preferentially bind to denatured proteins and unfolded intermediates to prevent their aggregation and trigger protein refolding, resulting in resistance to heat-induced apoptosis [89,90]. It could be shown that plasmonic photothermal therapy (PPTT) also initiates the expression of HSPs including HSP70 [91]. Therefore, it was speculated that the inhibition of HSP70 may improve outcome of photothermal therapy despite the reduction in membrane HSP70 as a targeting structure for nanoparticles. Ali *et al*. [92] conjugated gold nanorods with inhibitors directed against members of the HSP70 family (Quercetin) to evaluate their potential in sensitizing tumours to PPTT. Incubation of tumour cell lines (HSC, MCF-7) with AuNRs, PPTT displayed a low percentage of apoptotic cells, which was comparable to that of control cells. This disappointing result was attributed to high intracellular HSP70 levels that protect tumour cells from apoptosis. A knock-down of HSP70 by siRNA resulted in a significantly increased apoptotic cell death by PPTT. These results demonstrate a key role for intracellular HSP70 in the development of resistance of tumour cells to PPTT. Interestingly, even HSC cells that are overexpressing HSP70 could be sensitized towards PPTT after application of Quercetin-decorated AuNRs [92]. The results clearly demonstrate that a combined approach consisting of HSP70 targeting and inhibiting HSP70 function can significantly improve outcome of PPTT.

Another possible application of AuNPs is the radiosensitization of tumour cells [93] (figure 3). Owing to their high X-ray absorption abilities, AuNPs could increase dose deposition within target volumes even at relatively low concentrations [94]. A combination of HSP70-targeting AuNPs with radiotherapy could significantly increase this latter effect.

In conclusion, approaches based on NPs that aim to target membrane HSP70 on tumour cells might provide a promising strategy to significantly increase the specificity of tumour targeting and therapeutic potential. Furthermore, due to physico-chemical properties of the applied materials (i.e. Fe, Au, etc.), NPs also can be used for multi-modal anti-cancer therapy. Magnetite particles could be used in combination with hyperthermia, while AuNPs could be implemented in photothermal and radiation therapy. As demonstrated by Cui *et al*. [95], an improved drug delivery could be achieved by loading FE-containing functionalized magnetic particles with chemotherapeutics.

## 3. Conclusion

Depending on its intracellular, membrane or extracellular localization, HSP70 fulfils a variety of different functions in tumour cells. On the one hand, elevated cytosolic and membrane HSP70 levels mediate therapy resistance and thus contribute to the aggressiveness of tumour cells. On the other hand, extracellular and membrane HSP70 can stimulate the immune system and serve as a tumour-specific target. In this study, different approaches are discussed that use membrane HSP70 as a tumour-specific target for HSP70-functionalized nanoparticles of different qualities for imaging and combined anti-tumour therapies.

## Data accessibility

This article has no additional data.
